# Explaining of existing challenges of community-based undergraduate nursing education in Iran: a qualitative study

**DOI:** 10.1186/s12909-023-04484-x

**Published:** 2023-07-04

**Authors:** Foroozan Atashzadeh-Shoorideh, Arezoo Zeydani, Meimanat Hosseini, Sima Zohari-Anboohi

**Affiliations:** 1 Department of Psychiatric Nursing and Management, School of Nursing and Midwifery, Shahid Beheshti University of Medical Sciences, Tehran, Iran; 2 Student Research Committee, School of Nursing and Midwifery, Shahid Beheshti University of Medical Sciences, Tehran, Iran; 3 Department of Community Health Nursing, School of Nursing and Midwifery, Shahid Beheshti University of Medical Sciences, Tehran, Iran; 4 Department of Medical Surgical-Nursing, School of Nursing and Midwifery, Shahid Beheshti University of Medical Sciences, Tehran, Iran

**Keywords:** Community-based, Education, Nursing, Undergraduate

## Abstract

**Background:**

The education of nursing students should be such that the health needs of the community are met, but in Iran, due to some problems, students do not receive such education. Therefore, the present study was conducted to explain the existing challenges of community-based undergraduate nursing education in Iran.

**Methods:**

Ten individual semi-structured interviews were conducted with the faculty members and nursing specialists in this qualitative study. Eight focus group interviews were conducted to the nurses and nursing students using a purpose-based sampling method in 2022. The interviews were recorded and transcribed and then content analysis was done by the Lundman and Granheim method.

**Results:**

Five themes were obtained from the analysis of participants' responses, which include "weakness in community-based nursing education and curriculum", "treatment-oriented health system and education", "defect in the infrastructure and basic structures of community-based nursing education", "weakness in the implementation of community-based nursing education" and "weakness in the stakeholder engagement and cooperation of interested organizations".

**Conclusion:**

Interviews with the participants provided a vision of the challenges of community-based nursing education so that the reviewers of the undergraduate nursing curriculum in the ministry and nursing schools, educators, policymakers and nursing managers can use the results of the present study to improve the quality of education and the effective use of nursing students in responding to the community’s needs and provide a proper context for improving students' learning.

## Introduction

Community-based education includes learning activities in which the community is used as a primary learning setting, in which students, nursing educators, community members and representatives from other sectors actively participate in the educational experience [1]. This program can improve the quality of life of individuals, families and communities and reduce health care costs [2]. Community-based education includes the integration of education and practice in the community for learning [3]. It is in such a way that it prepares students to accept their professional roles in the community by providing relevant education and creating learning opportunities. By receiving the necessary education, nurses can provide services in complex situations of the community [4]. In the meantime, a number of countries such as South Africa, the United Kingdom (England) and America follow the community-based education program [5–7]. Due to the expansion of health care in the community, the demand for capable nurses to work in the health setting of the community has been increased, and the focus of attention has been changed from health care in the hospital to health care in the community [8].

Educational systems are facing various challenges. The professional competency of graduates, and the development of the quantitative dimensions of education, require exact planning and attention to the quality of educational programs and awareness of new methods in the education process [9]. Education should occur in the context of community and close encounters with the clients and their problems to achieve deep learning. Still, community-based nursing does not have a proactive role in the undergraduate nursing curriculum in Iran. Nursing undergraduate students do not receive the necessary education to play a professional role at the community and health service provider centers. As a result, they cannot cover the goals of community-based nursing, which is to maintain and promote the community's health. They cannot operate effectively in the community [10].

The results of many comparative studies aimed to compare Iran's undergraduate nursing education program with the countries of the world [11], developed and developing countries [12], Jordan and Turkey [13], Australia [14], South Korea [15], California [16], America [17], China [18], Japan [19], Singapore [20, 21], Malaysia [22], England and Canada [23, 24] have shown that the present nursing education program in Iran has defects and weaknesses and needs to be revised.

The results of Barasteh et al.'s study on the future challenges of Iran's nursing system showed that considering Iran's demographic changes and moving towards the tsunami of aging and chronic diseases, it is necessary to modify educational and service policies according to the community’s needs. In the country, nursing services are mainly provided at the level of hospitals, and the activities of nurses do not meet the community’s needs, because nurses do not receive education according to the community’s needs [25]. Also, the conditions of undergraduate nursing education in Iran are that students are mostly trained to serve in hospitals and the training provided is hospital-based and emphasizes treatment. At the same time, paying attention to the health needs of the community and empowering students to provide services in the community is very low, while many of the existing problems can be solved by providing services in the community [12, 16, 26, 27]. Therefore, due to the high importance of community-based nursing education and the failure to train nurses to respond to the community’s needs, the researchers decided to conduct the present study to explain of existing challenges of community-based undergraduate nursing education in Iran so that through it they can identify the problems and barriers for achieving the goal.

## Methods

### Design/Participant

In this qualitative study, 42 nursing faculty members, nursing policymakers, nurses, and nursing undergraduate students from different universities, cities, and provinces throughout Iran were selected through purposive sampling. To explain the present situation of community-based undergraduate nursing education, the approach of manifest and latent conventional content analysis and Lundman and Graneheim method was used [28]. The inclusion criteria for the faculty members included membership in specialized nursing groups, teaching and training experience for at least three years. The inclusion criteria for students was semesters four to eight of nursing, and nursing graduates were a maximum of two years after completing the bachelor's degree. The interviews were conducted in the semi-structured method after obtaining informed consent. Therefore, ten individual interviews were conducted with the faculty members, two focus groups with the nurses and six focus groups with the nursing students. Based on the participants' agreement, the interview setting was determined at the place of study, workplace, or through audio and video communication on the social network (Skype, WhatsApp).

### Data collection

This research used semi-structured interviews and focus group discussions to collect data. The duration of individual and group interviews was between 60 and 90 min, and the time of supplementary interviews was between 30 and 60 min. The interview guide was prepared in advance and included three questions about the current status of community-based nursing continuing education: how do you see the quality of community-based nursing education in the current system? What are its positive and negative points? What knowledge and skills has the existing educational program helped you/students acquire? What is the gap in providing healthcare services at the community level? To gain a deeper understanding of the research topic, a number of probing questions were also asked the participants, examples of which included the following: describe your experiences in this field, how do you see the cause of this problem, explain more and give your experiences in this field, what do you mean? Please explain your experiences more clearly and precisely. At the end of the interview, all the participants were requested if they wanted to express a point or issue that was not discussed during the interview. The interviews ended after data saturation. After data saturation, three individual interviews and two focus group interviews were conducted to ensure that no other findings were added to the content analysis process and with this data collection method, data saturation was achieved. It should be noted that to avoid possible bias in the data collection process, the second author conducted all the interviews under the supervision of other authors, and the data were collected over one year.

### Data analysis

In this research, at the same time as conducting the interviews, data analysis was done using the Lundman and Graneheim method [29]. Therefore, after conducting each interview, the recorded information was listened to several times. Then the interviews were written word by word on paper and typed and reviewed again with the recorded information. Then the raw data was transferred to MAXQDA software version 2018, and the conventional content analysis method was used to analyse the data. In this way, to understand the importance of the interview, the transcripts were read several times. The meaning units were identified and summarized as codes. The extracted codes were based on semantic similarity in the subcategories, and the subcategories were sorted into the relevant categories based on commonalities and thematic. This process was done in several rounds of reading and a number of subcategories and categories were modified. To avoid possible bias in the analysis process, the correctional comments of all authors were used. In addition to the authors, the results obtained from the process of content analysis were reviewed by three nursing doctorate experts.

### Trustworthiness

In this research, the criteria of credibility, confirmability, dependability and transferability of Lincoln and Guba were used to ensure the trustworthiness and rigor of the data [30, 31]. In this research to fulfill the above items, from the methods of continuous engagement with the data, the corrective comments of the professors during the process of interviews and their analysis, review of the manuscripts by the participants, integration in the method of data collection in the form of individual interviews and group, the use of the opinions of three nursing doctorate experts, the use of appropriate samples, the simultaneous collection and analysis of data, and the use of a specific procedure for coding and data analysis were used.

## Results

There were 42 participants in this research. Table 1 presents the demographic data of participants.

**Table 1:** The demographic data of participants

Interview	Individual (10)	Focus group (8)	
Participant	Faculty member	Nurse	Student
Frequency	10	6	26
Gender	7 Women	4 Women	8 Women
	3 Men	2 Men	18 Men
Age range	36–90	23–26	20–27
Education level	PhD	Master	Bachelor of nursing
Clinical experience (Month & Years)	4 M -10Y	1 M-2 Y	-
Educational experience (Years)	3–67 Y	-	-
Scientific ranking	Instructor (2)	-	-
	Assistant professor (4)	-	-
	Associate professor (4)	-	-
Field	Nurse (9)	Nurse	Nurse
	Public health (1)		
Semester	-	-	4–7
Clinical department	-	Emergency (2)	-
	-	Critical care (3)	-
	-	Clinic (1)	-

The findings showed 84 Subcategories, 18 categories and 5 themes (Table 2).

**Table 2:** Subcategories, categories and themes obtained from content analysis of individual and focus group interviews

Theme	Category	Subcategory
1. Weakness in community-based nursing education and curriculum	1–1. Weakness in the community-based nursing educational content	- Inefficiency of community health nursing lesson content - Relative desirability of theoretical content and heading of undergraduate nursing lessons
	1–2. Weakness in community-based nursing curriculum	- Defects in school nursing education program - Defects in starting the nursing education program with healthy people - Starting the nursing education program with sick people - Defects in the localization of the nursing curriculum
	1–3. Weakness in community-based nursing education	- Inefficiency of community-based education - Inefficiency of community-oriented education - Trivialization of community-based education - Insufficient attention to prevention levels in nursing education - Low number of community health nursing course units - Weakness in educational justice with different status of current education implementation
	1–4. Inefficiency of community health nursing clinical education	- Defects in the practical education (internship) of newborn, women and parturition health for male students - Long duration of the community health nursing internship - Inefficiency of training students in comprehensive health service center - Lack of difference and diversity in health and medical and surgical nursing internships - Repetitive and everyday activities during the internship - Focus only to the vaccination unit during the internship - Lack of learning load in community health internship for students - Waste of student's time in community health nursing internship - Weakness in community health internship education for students - Defects in training of nursing care in the community - Inadequate supervision in community health nursing internship - Lack of student education in the community settings (homes, factories, schools, welfare, nursing homes, kindergartens)
	1–5. Limited human resources and community-based education facilities	- Inadequate equipment, educational and welfare facilities for students in comprehensive health service centers - Lack of community health nursing professors - Lack of educators in community health nursing internship - Limited health service centers and community settings - Lack of necessary powers of professors for training in comprehensive health service centers - Poor education due to the presence of a large number of nursing students in a comprehensive health service center - limited opportunity for practical learning of students due to their high number in a comprehensive health service center
	1–6. Problems in community-based skill empowerment of students	- Deficiency in creating student skills to work in the community - Defects in building self-confidence of students to work in the fields of community
	1–7. Deficiency in the matching of theoretical and practical community-based education	- Inconsistency of theoretical education with its internship - Inconsistency of theory and practice with the situation and needs of community - The gap between theory and clinical practice - Using translated foreign references for teaching
2. Treatment-oriented health system and education	2–1. Hospital-centered and treatment-centered in the health system and education	- Patient-centered educational approach of nursing educators - The predominance of the health system's attention to hospital-oriented and treatment-oriented education - Disease-oriented approach to nursing education - Deficiency in community health provision due to hospital-based education
	2–2. The predominance of the clinical-oriented perspective	- The clinical-oriented perspective of nursing students and educators - The clinical-oriented perspective of policy-makers - More students' interest to treatment activities than health
3. Defect in the infrastructure and basic structures of community-based nursing education	3–1. Weak support and legitimacy of the community-based education program	- Weakness in the support of faculty members for health-oriented education - Deficiency in legal support for students and educators to work at the community level - Weakness in the support of policymakers for activities at the community level
	3–2. Defects in the position and role of community-based nurses	- Lack of position and organizational level for community health nurses - Deficiency in the definition and clarity of the position of the nurse in the health structure and society - Limiting the definition of the position of a nurse to the hospital setting - Policymakers' opposition to clarifying the position of nurses in community - Defects in defining the role of nurses in community - Failure to pay attention to the nurse's role in communicable and non-communicable diseases - The lack of clarity of duties and the interference of the nurse's role with the environmental health expert - Ignoring the nurse's role in prevention levels - Defects in employing nurses by organizations - Defects in the existence of nursing models in community
	3–3. Defects in the infrastructure of community-based education	- Defects in the entry of nurses into the structure of the health system - Defects in the availability of community-based education structure - Defects in the infrastructure of the nurse's position in the community setting - Deficiency in issuance of nurse’s employment permit in the community - Deficiency in education of the third level of prevention due to lack of patient referral system after discharge
	3–4. Defects in the establishment of nursing clinics	- Defects in the development of regulations for nursing clinics - Problems in setting up a health-oriented clinic
	3–5. Defects in the trust, awareness and acceptance of nurses in the community by the people	- People's ignorance of the role of nurses in community - People's ignorance of the services provided by comprehensive health service centers - People's lack of trust in the ability and knowledge of nurses - Defects in the acceptance of community health nurses by the community
4. Weakness in the implementation of community-based nursing education	4–1. Weakness in the home visit, home care and patient education	- Procedure-oriented home care services - Weakness in the practical implementation of home visits - Weakness in the practical implementation of home care - Training to the client/patient superficially
	4–2. Intangibility of lessons and defects in the implementation of the community-based program	- Problems in the tangibility of community health lessons in implementation - Limitation in operationalizing community health lessons - Defects in the implementation of community-based education and its implementation
5. Weakness in the stakeholder engagement and cooperation of interested organizations	5–1. Weakness in the cooperation of the personnel of comprehensive health service centers with educators and students	- Not allowing vaccination to the student - Resistance and opposition of comprehensive health service centers to the entry of nurses - Weakness in the cooperation of health center and school staff with nursing educators and students - The existence of resistance and many bureaucracies to coordinate the entry of students into the settings of community - Communication gap between nursing schools and comprehensive health centers
	5–2. Problems in the attitude, behavior and mutual performance of the health care centers officials and students	- Inappropriate behavior of officials of comprehensive health service centers to the students - Occupational abuse of students by health care centers - The negative attitude of employees towards the presence of students and professors in internship settings - The negative attitude of students towards the misuse of them by the health care centers

The themes generally refer to the problems and barriers of nursing education and as well as it raises the challenges in the health system, the ministry, the policymakers, the university, the faculty, the program and its implementation, officials, educators, students, health care training centers, community and people, other organizations and health service provider teams. An example of each theme and quotes related to participants' experiences in individual and group interviews are given:

### The first theme: Weakness in community-based nursing education and curriculum

- This curriculum is not localised to Iran; we took it from other countries, saw what they teach and their books, brought them here and wanted to implement them (Participant 8).

- We are extremely weak in the field of community-oriented education (Participant 1).

### The second theme: Treatment-oriented health system and education

- You can see that our health system gives more importance to treatment; I know that 60 to 80% of the gross domestic product is spent on treatment, while in developed countries; it is spent on prevention (Participant 2).

- All the students and nurses showed particular special interest only in doing some clinical work (Group 1 of nurses).

### The third theme: Defect in the infrastructure and basic structures of community-based nursing education

- Our graduate nurse completes about 5 to 6 units of community health lessons and can easily enter social fields. However, we still have not considered nurses’ rank and professional position in the community (Participant 9).

- Let's look at the Ministry of Health's 2012 diabetes guideline program, in which the role of the nurse is defined, but this nurse has not yet entered the health structure, a position at the first level of prevention has been defined, but not yet entered (Participant 6).

### The fourth theme: Weakness in the implementation of community-based nursing education

- Home care is the name of one of the units, but students only take exams and do not have practical work at home (group 2 of nurses).

- How to implement community-based education in the environment is essential. We have problems (Participant 3).

### The fifth theme: Weakness in the stakeholder engagement and cooperation of interested organizations

- In health centers, when we enter and want to work in practice, environmental health workers do not allow us to enter this area and say that you are interfering in our work (Participant 2).

- We went to the center for one or two days, and it mainly was abuse; for example, they told us to sit and sort the files, which was not an excellent job at all and it was not related to us, and it was more abuse than learning (Group 1 of students).

## Discussion

The results of this research include 5 themes "weakness in community-based nursing education and curriculum", "treatment-oriented health system and education", "defects in the infrastructure and basic structures of community-based nursing education", "weakness in the implementation of community-based nursing education", and "weakness in the stakeholder engagement and cooperation of interested organizations".

In line with the present study’s findings, previous studies showed that the content, education, and program of nursing education provided are weak in terms of being community-based. Because it is incompatible with the educational goals, philosophy, mission, and community’s needs, Iran’s cultural and local conditions do not match and need to be revised. This issue has also caused a gap between theory and practice, paying attention to it reduces the gap between nursing theory and practice. Focusing on the lessons that suit the community’s needs and its localization is one of the suggestions presented in different studies [2, 27, 32, 33].

Hosseinnejad et al. (2022) showed in a systematic review that nurses face various challenges in providing community-based care. In their study “Infrastructural challenges” such as insurance coverage and financing, defects in implementation and weakness in interdisciplinary cooperation, and “cultural challenges” such as public distrust and negative attitude towards the capabilities of non-medical professionals in providing prevention and health services were obtained. In addition, “educational challenges” such as students' poor understanding of community health nursing courses, considering community health nursing internships as futile, and poor efficiency of community health internship programs, were obstructed. The last theme “policy challenges in nursing” such as failure to determine the position for providing nursing services in the community and health system, lack of clear job descriptions for community-based nurses, and so on were emphasized in their study [32]. Most of their findings are in line with many of the results of the present study. It seems that a large part of the problem is related to the infrastructure, which due to the lack of a community-oriented perspective of the officials and the existence of a treatment-oriented perspective, no action has been taken for it, and correcting it; educational problems can be solved to a large extent.

In line with our findings, such as "weakness in the community-based nursing educational content", "limited human resources and community-based education facilities", and "weak support and legitimacy of the community-based education program", Kaye et al. (2011) have shown that there are limitations in human resources, facilities and support and deficiencies in the community-based educational program content [34]. In a study conducted by Nuuyoma et al. (2022), the limitation of human resources and facilities was mentioned as a challenge of community-based education [35]. Therefore, nursing schools should consider community-based educational programs and provide facilities and resources to promote educational benefits because community-based education requires a consistent program design to achieve positive outcomes. It is necessary to consider the challenges mentioned in the nursing curriculum to be implemented well.

In line with the present study’s findings, such as "problems in community-based skill empowerment of students", the results of some studies showed that students' employment is only in clinical environments such as hospitals. There is a lack of specialized and suitable positions for nursing graduates in health centers and at the community level, hindering them from acquiring the necessary skills to provide health care is provided by nursing graduates [36–38]. Also, Karimi et al.'s study (2013) showed that nursing graduates could not provide health care outside the hospitals [33]. Students do not acquire the necessary skills due to the lack of positions for nurses in the community and the lack of activity in it. Therefore, one of the ways to empower students is that they must have work experience at the community level, and this issue requires defining a position for them.

The results of the study by Nuuyoma et al. (2022) in line with the findings of the current study "defects in matching theoretical and practical community-based education" showed that there is a gap between theoretical and practical education as one of the challenges of community-based education and placing students in the community helps reduce this gap [35]. Karimi et al. have mentioned that inapplicability is one of the problems of community-based nursing education from the students' perspective. This seems to be reflected in the employment of most nurses in treatment centers such as hospitals, regardless of their specific position in health care centers. As a result, this issue causes, for example, students not to have a correct understanding of health lessons and consider them useless [33, 39].

In line with the findings of the present study, "weakness in the home visit, home care and teaching to the patient", the results of the study by Nikbakht Nasrabadi et al. (2016) showed that home care and home visits are associated with challenges such as lack of resources, lack of required infrastructure such as lack of insurance regulations, improper performance and indifference towards it, therefore, if clients and families have problems, they have no choice but to go to medical centers. They recommended that a system should be established to provide healthcare services at home due to the increase in old age in Iran and healthcare costs, [40]. The results of the study by Khaleghparast et al. (2018) showed a weakness in the field of patient education. This weakness was caused by too many tasks, lack of time and sufficient personnel, lack of interest, motivation, and desire for education, not knowing the duty, and not knowing about the things that should be taught to the patient [41]. Therefore, it seems that the weakness in home visits and home care, and patient education is caused by the weakness of the infrastructure, insurance, and insufficient resources, and their strengthening can improve the implementation capability of the above.

In line with the study of Mohammadi et al. (2014) findings of the present study showed "hospital-centered and treatment-centered in the health system and education" and "the predominance of the clinical-centered perspective" are the most critical challenges of nursing education which shows the predominance of the biomedical paradigm in the educational program. They have mentioned that attention to disease and treatment is dominant in education compared to the health paradigm [42], and this importance should be considered in the existing curriculum.

The findings of Hajbaghery et al.'s study showed that the nursing curriculum in Iran is focused on disease and treatment and is taken from nursing courses and lessons from foreign countries. For this reason, it is not appropriate for the community’s needs [16]. Also, based on numerous studies, the weak presence of community-based care in nursing education, the focus on hospital care, and the focus of nursing education on the clinical in Iran have caused most nursing schools to train their students to play the traditional nursing role. At the same time, the community needs the education of nurses based on holistic views [26, 33, 43]. Hosseinnejad et al. (2022) have stated that currently, the focus of the health system is on disease-oriented approaches instead of prevention-oriented ones, and this issue is an essential reason for high costs in the treatment sector and causes problems in allocating resources to nurses in the health field [32]. The study’s result by Karimi et al. (2013) showed a weakness in nursing students’ community-oriented and holistic attitude. Most students understand the role of nurses only in the hospital and are clinical-oriented in practice, which conflicts with the philosophy of the nursing curriculum [33].

The findings of the current study include "weakness in community-based nursing education", "defects in the position and role of community-based nurses", "defects in the infrastructure of community-based education", "defects in the trust, awareness” and “acceptance of nurses in the community by the people" in line with Heidari et al.'s study (2017), indicates the existence of challenges in community-based education. They considered the infrastructure defects, position and job opportunities for community-based nurses, and the lack of community's readiness to receive community-based nursing services as one of the critical challenges of community-based education [2]. Hosseinnejad et al. (2022) have also confirmed the lack of the position and role of the community-based nurse in their study [37]. The healthcare system’s current infrastructure is not suitable for first and third-level prevention. Nursing graduates are mostly trained to do clinical work and work in hospitals, job opportunities for community-based nurses are limited, and the community is not ready to receive community-based nursing services. Therefore, it is necessary to create and modify the infrastructure to facilitate the entry of community-based nurses into the community and its training [2]. One of the reasons for the lack of trust, awareness, and acceptance of nurses in the community is public mistrust and negative attitude toward the capabilities of non-medical professionals in providing prevention and health services [26, 44]. By creating job opportunities for community-based nurses [37] and the positive consequences of the nurse's presence in the community and its abilities, the community’s trust can be gained over time [2].

In line with the findings of the present study, "defects in the establishment of nursing clinics", the results of the studies showed that health and treatment managers should support nurses in establishing health-oriented nursing clinics [33, 45, 46], and regarding the setup of home visit centers using the health promotion approach to the help of nurses takes action [47, 48].

In line with the findings of the present study, "weakness in the cooperation of the personnel of comprehensive health service centers with educators and students", "defects in the position and role of community-based nurses" and "defect in the infrastructure of community-based education", the findings of the study by Rahnavard (2018) raised the existing challenges of community-based nursing education in Iran, inconsistency and inability in team working, lack of cooperation, defects in the position of community-based nurses, and defects in the necessary infrastructure for the community-based nurse are among the items mentioned in the study, for example, the nurse does not have the position in the family physician's team. In addition, community-based nursing does not have a role in Iran's undergraduate nursing curriculum, and the education of students does not take place in the heart of the community and close confrontation with clients and their problems. There is no necessary infrastructure, for example, one of the critical infrastructures is creating a culture in health care centers, policymakers, and the community. If community-based nursing is understood, it will be used effectively and optimally, leading to positive results [10]. In addition, the findings of the review study by Bvumbwe and Mtshali (2018) in South Africa showed challenges in cooperation, partnership, infrastructure, and resources. They considered an extensive partnership investment, collaboration, and teamwork necessary [49].

The findings of Barasteh et al.'s (2021) study about the future challenges of nursing in Iran's health system showed that in the field of legislation, cooperation, and communication with other institutions, the inadequacy of professional development with social needs and the role of community-based nurses and defects in nursing education according to the community’s needs, there are challenges. These findings are consistent with the results of the current study, including "weakness in the cooperation of the personnel of comprehensive health service centers with educators and students", "weak support and legitimacy of the community-based education program", "defects in the position and role of the community-based nurse", and "weakness in community-based nursing education. Considering Iran's demographic changes and moving towards a tsunami of aging, chronic diseases, and related disabilities, it is necessary to modify educational and service policies according to the community. Still, nursing services are mainly provided at the level of hospitals in Iran. The activities of nurses do not meet the needs of the community. Unfortunately, health-oriented centers in Iran's health system are limited, and the existing centers are mainly treatment-oriented. Besides, extensive cooperation with the government organization and other related institutions (welfare organizations, municipalities and radio, and television), and legal support of students and educators are needed to ensure their safety and community members so that the education of students at the community level is done well [25].

In line with the present study’s finding "intangibility of lessons and defects in the implementation of the community-based program", some studies have shown the lack of application of some of the contents of theoretical lessons in internship environments and subsequently created a gap between theory and practice. This issue has been caused by what is read in theory is not tangible in practice, and the program’s implementation faces problems [2, 32, 33]. It seems that if the curriculum is modified based on the structure and health needs of the community, this problem will be mainly solved.

In line with the findings of the present study, "problems in the attitude, behavior and mutual performance of the health care centers officials and students", Jafarian et al. (2020) showed that students in practical training environments face many challenges, such as violence, fear and anxiety, humiliation and blame, lack of support and inappropriate behavior of employees. They have recommended that due to the existence of injustice in practical training environments, for example, the use of students as section employees, coercion, and the use of physical and psychological violence against them, to suppress such injustices, fundamental reform in the organizational culture educational health care centers should be done. More coordination should be done by the managers of colleges and existing centers at the community level [50].

## Suggestions

According to the findings of the present study, it is suggested that more studies should be done in the field of a clear explanation of the role of community-based nurses in community settings and separate them from the duties and functions of public health and environment experts. Clarifying the position, description of responsibilities, and the organizational level of community-based nurses, identification of endemic diseases, and the health needs of different provinces and regions of the country are critical. Suggestions for including educational content in the undergraduate courses program of each university, identifying strategies to attract the cooperation of different professions and centers, and providing an operational plan to expand the community-based education infrastructure is essential.

## Strengths

The strength of the present study is a large sample size with maximum diversity. Different nursing educational groups were used in order to increase the generalizability of the findings and the richness of the information.

## Limitation

The limitation of the present study was that some participants refused to give complete information or self-censored, which was solved to a large extent by assuring the confidentiality of the interviews and asking probing questions.

## Conclusion

The results of the present study showed that the current undergraduate nursing education has weaknesses and defects in terms of being community-based. Also, interviews with the participants provided a vision into the challenges of community-based nursing education so that the reviewers of the undergraduate nursing curriculum, educators, policymakers and nursing managers can use the results of this study to improve the quality of education and provide a proper context for enhancing students' learning.

## Ethical considerations

All samplings were done after obtaining the approval of the research ethics committee of Shahid Beheshti University of Medical Sciences (approval ID: IR.SBMU.RETECH.REC.1399.363) and providing the necessary explanations about the intended research to the participants and after obtaining their consent. The time and setting of the interview were determined based on the opinions and agreement of the participants. In the interview sessions, one of the researchers emphasized the confidentiality of information, obtaining informed consent and maintaining a private environment for the participant. They were also informed that they could exit the study at any time. The research was conducted in accordance with the principles of the Declaration of Helsinki.

## Data Availability

All data generated or analyzed during this study are included in this manuscript.
